# Developing a three-dimensional narrative to counter polio vaccine refusal in Charsadda

**DOI:** 10.7189/jogh.10.021301

**Published:** 2020-12

**Authors:** Sheraz Ahmad Khan, Muhammad Ashfaq, Ayaz Ayub, Ayisha Jamil, Jehan Badshah, Izzat Ullah, ASM Shahabuddin, Faraz Khalid

**Affiliations:** 1 Department of Health, Government of Khyber Pakhtunkhwa, Peshawar, Pakistan; 2 World Health Organization (WHO), Charsadda, Pakistan; 3 Institute of Public Health and Social Sciences (IPH&SS), Khyber Medical University (KMU), Peshawar, Pakistan; 4 ICU Healthcare, Peshawar, Pakistan; 5 Implementation Research and Delivery Science Unit, Health Section, Programme Division, United Nations Children's Fund, New York, USA; 6 United Nations Children's Fund, Islamabad, Pakistan

## Abstract

**Background:**

Endemic polio in Pakistan is threatening the Global Polio Eradication Initiative (PEI). In recent years, vaccine refusals have surged, spiking polio cases. The current study was conducted to understand the ethnic, religious and cultural roots of vaccine refusals in Charsadda District and explore the remedial options.

**Methods:**

We conducted 43 in-depth interviews with parents who had refused polio vaccines for their children and the PEI staff. Interviews were audio-recorded, written in verbatim and analysed with Atlis.ti. We conducted a thematic analysis of our data.

**Results:**

The fear of American and Jewish conspiracies was the primary cause of vaccine refusals. Militant groups like Tehrek-i-Taliban Pakistan capitalised on this fear, through social media. The Pashtun ethnic group considers itself at the centre of conspiracies. They are suspicious of mass investment and mobilisation behind the polio campaign. Our respondents feared that polio vaccines were making children vulgar. They also feared a reduction in the male to female ratio in childbirth. In Pashtun communities, the iconic conventional community gatherings [“Hujras”] are being replaced by provocative digital Hujra [social media], which the PEI and the Government of Pakistan (GOP) are failing to influence or regulate. The PEI uses the misleading term 'religious refusal'. Some factions in the clergy are maligning people from vaccinations, but not through religious dictum. The anti-state elements have stirred sentiments to weaken the state initiative. Fear of adverse effects, attitudinal barriers of health care providers, unmet basic needs and alleged haram composition of the vaccine were among the reasons for vaccine refusals. The PEI needs to revise its misleading nomenclature and ensue open discussion to dispel the myths of infertility, vulgarity and gender ratio related to the vaccines. Simultaneously, the GOP should stop disinformation on social media and rebrand polio vaccination with popular initiatives like the government-sponsored health insurance schemes.

**Conclusions:**

The ethnic, cultural and religious dispositions of community members shape polio vaccine refusals in Charsadda District, in different ways. In synch with existing conspiracy theories and medical misconceptions, these three factors make refusals harder to counter. Awareness campaigns with content addressing these three dimensions can improve the situation.

Polio eradication is a public health challenge. With a sharp rise in polio cases in 2014, Pakistan's situation was declared as “public health emergency of international concern” by the International Health Regulations and Emergency Committee of the World Health Organization (WHO). Polio endemic in Pakistan was labelled as a threat to the international polio eradication efforts [1].

The Global Polio Eradication Initiative (GPEI) is faced with numerous challenges in Pakistan, including illiteracy, misinformation, medical misconceptions and a weak health care system [2]. These challenges are more pronounced in the Pashtun belt than the rest of the country [2,3]. The Pashtun ethnic group makes eight per cent of Pakistan's population but reported 77 % of polio cases in 2011 [4]. These cases could be attributed to the rising trends in vaccine refusals that followed the insurgency and counter-insurgency operations in the Pashtun belt [4].

In the Pashtun belt of Pakistan, district Charsadda serves as an active hub of nationalist politics and religious ideologies [5,6]. Charsadda has seen violence against polio vaccinators [7], and the government is taking punitive actions to vaccinate children.

The district has an ongoing Expanded Programme on Immunisation (EPI) and the Polio Eradication Initiative (PEI). The PEI is facing persistent vaccine refusals. Around 2000 vaccine refusals are reported with every National Immunization Day (NID) from the area.

Evidence suggests that a counter-narrative for vaccine refusals, considering local experiences and concerns, should be adopted [8]. To inform such a counter-narrative, the current study was conducted at Charsadda, to explore the cultural, religious, and ethnic underpinnings of vaccine refusals and suggest reformative actions.

## METHODS

We conducted our research with a relativist ontological and interpretive epistemological position, using a case-study methodology and in-depth interviews as the data collection method. We took district Charsadda as a case-study, as the district has profound religious and political influence over the entire Pashtun belt of the country.

We conducted 43 in-depth interviews with community members (n = 23) and the PEI staff (n = 20). At the community level, we interviewed residents of district Charsadda who had refused polio vaccines for their children. A researcher in our team (MA) who worked with the PEI, helped with recruiting the participants. Among the PEI staff, there were two subgroups: frontline PEI workers and top management of PEI.

We interviewed fifteen front line polio workers, including five union council polio officers, five union council communication officers, and five religious support persons. We conducted four interviews with the upper tier of PEI leadership, including the provincial team leader for PEI at WHO, provincial team leader for UNICEF, and deputy director EPI Khyber Pakhtunkhwa. Two interviews were conducted with well-versed, educated persons, affected by polio in childhood. The initial interviewees were identified by the team member (MA), while the rest of the respondents were selected through snowballing.

At the community level, we conducted fifteen interviews with male respondents and eight interviews with female respondents. Fewer interviews were conducted with female due to cultural barriers. After doing a total of 43 in-depth interviews, we observed saturation in our data and stopped further data collection.

Our research team consisted of members from the local Pashtun ethnic group. The researchers knew the local ethnic and social construct, which helped with conducting the interviews. However, it added some complexity to our research, as frequent discussions were needed among the team members, to ensure self-awareness and reflexivity in our work.

Our team included members with medical, nursing and social sciences background, enabling us to respond to the interviewees' concerns, follow the leads during the interviews and reflexively check our interpretation of data with other team members.

Similarly, we had both male and female interviewers. This team composition facilitated interaction with the respondents, especially the female, at the community level. It also brought a gender lens to our data collection, analysis and interpretation processes.

### Data collection and analysis

All 43 interviews with the participants were conducted in their natural settings. Before starting our study, we attained ethical approval from the ethical review board of the Health Services Academy (HSA), Islamabad. After ethical clearance, we attained an administrative approval along with signing a memorandum of understanding with the district EPI/ PEI officials. For each interview, we attained informed consent from our respondents. With the community members, consents were verbal as they were not comfortable with written consent due to fear of administrative action. From PEI staff, we took written consents.

We conducted all interviews in the local language, ie, Pashtu. With prior permission of participants, we audio-recorded the interviews. Interviews were written in verbatim. The Pashtu scripts were translated into English and then back-translated to ensure accuracy. We kept both the audio-files and the transcripts completely anonymous and secure.

We conducted a thematic analysis of our data. Initially, we conducted open coding and then categorised similar codes under related themes. We used “Atlis.ti” software for our qualitative data management.

## RESULTS

We assembled our study findings under five themes. Theme-1 describes different reasons for vaccine refusal. Themes 2-4 represent respondent's perspectives on how the ethnic, cultural, and religious notions influence their predisposition to vaccine refusals. Theme-5 presents stakeholders' views on steps to convert vaccine refusals to acceptance.

### Theme 1: Reasons behind vaccine refusals

The old conspiracy theory of polio vaccination as a Jewish plot is still at the heart of vaccine refusals. One of our respondents said: “*Many people believe that the polio vaccination campaign is a conspiracy of Jews. In the Municipal Committee-4 area, there was a person who was a case of chronic refusal. He was imprisoned because of his refusal, but still, he did not agree to vaccinate his children.*”

Fear of infertility was among the leading causes of vaccine refusals. One of our respondents on this account said: “*My husband is strongly against vaccination. He worries about the elimination of our future generation; everybody does. We all want our children to have kids.*” On a related note, another respondent said: “*it causes infertility in women. Also, it causes the birth of more female children compared to male babies*.”

There is a trust deficit at the community level. One female respondent said: “*Why should we trust our government which never brings us requirements of our living like sugar and flour, and which is unable to employ us, yet has endless resolve and resources for the polio vaccination campaign. What is so special about it?*”

In other instances, the trust deficit arises from questioning the credibility of vaccinators. A community member said, “…*there are rumours that the team members are just filling the bottles from water, for the sake of their daily remunerations.*”

### Theme 2: Pashtun ethnicity and vaccine refusals

The Pashtun ethnic group considers itself at the centre of national and international conspiracies. One of our respondents said: “*Pashtuns face numerous challenges everywhere, all the time and across the globe due to some facts. First, they are brave; second, they accept challenges; third, they never let down their turbans concerning honour. So, we believe that a polio vaccine is a tool for devastating Pashtuns entirely.*”

When asked if ethnic ideology shapes the community's behaviour, one respondent said: “*Yes, Pashtuns feel discriminated, and they are suspicious of the state's mass investment and mobilisation behind the polio campaign. They are reluctant.*”

Another respondent mentioned that administrative action is further stoking hatred. He said: “*Sometimes the government imposes section-144* [ban on free movement and aggregations] *due to polio vaccination. The government knows that the Pashtuns would not allow such objectionable and ineffective drops for their children. Now they are using police, but how can they force us, these are our children, and we will decide what is best for them.*”

### Theme 3: Religious considerations and vaccine refusal

The PEI uses the term' religious refusal', which is misleading. One of our female participants said, “*Islam does not forbid us from polio vaccination, but the imam does…for the same reason that America is manufacturing it, and it may be a conspiracy. All our men have the same concept.*”

Those with a more profound attachment to religious circles have a deep hatred for foreign forces. One family labelled as religious refusal, when asked about their cause for refusal, said that: “*When America is killing our officers and our elders, how should we presume that what they are sending for our children is safe and harmless? It is a conspiracy plot to make our generations shameless and vulgar.*”

Regarding the clergy's influence, one participant said: “*It is better to persuade one religious scholar rather than the persuasion of hundreds in a locality. Religious scholars will convince people to feed the vaccines. Masses will accept the Maulvi's instructions willingly.*”

In some instance, the religious beliefs are manoeuvred to make people refuse vaccination. One respondent said, “…*religion is used as a political tool, for example, with regards to vaccine refusals. Who knows, if anti-state elements have stirred religious sentiments against polio vaccines on purpose. Might be! It looks like that.*”

### Theme 4: Cultural considerations and vaccine refusals

Our respondents believed that the vaccination drive did not conform with the conservative Pashtun culture. In the words of one respondent, “*These vaccines are affecting our culture, making our children vulgar and disrespectful to their elders. It is a plot to control our population growth.*”

Similarly, keeping in view the patriarchal structure of the society, there is a natural liking for male children, and hence this cultural preference plays into vaccine refusals. One respondent said: “*These drops affect [decrease] the ratio of male offsprings compared to female. The drops alter the genetic matter [sperm and ova]. Then, the element of modernism and increase of obscenity [vulgarity] are among the major effects of these vaccinations. Therefore, we keep our children away from polio vaccinations*.”

Another peril of the Pashtun culture is that women in rural areas are impossible to reach, causing an information barrier. When asked about the role of Hujra in addressing the issue of vaccine refusals, one female respondent said: “*Hujra is the place of finding solutions to common problems…If the polio teams come to Hujras to converse with the male family heads, it will surely bring benefit.*”

The state is resorting to force for polio vaccination. One frontline polio worker labelled use of force as detrimental. He said, “*I would advise against the involvement of administrative authorities in vaccination…vaccinators are now accompanied by police or mayor. This stringent attitude towards people will surely increase their disapproval.*”

The punitive actions are further stoking hatred against polio vaccination. One respondent said, “*using force or police against the families who refuse vaccination is leading towards the genesis of more and more doubts and hatred for our polio teams. It creates a very hostile work environment for us in the subsequent campaigns.*”

### Theme 5: Ideal steps to enhance vaccination uptake

The overwhelming message in this theme is that the community has unmet information needs. One front line worker said, “…*our government is lagging in the awareness part. Why doesn't the government make it compulsory on all television channels to have PEI and EPI related awareness messages?*”

The information flow needs improvement in content and quantity. Regarding the content, a community member said, “*once the campaign starts, we come to know* [from banners] *that polio vaccination has started. So, the current campaign material tells about the campaign-dates only.*”

About the content of information rolled-out, a frontline worker said: “*I suggest and emphasise the government to spread more information regarding polio vaccines' composition and ingredients.*”

Similarly, a frontline worker pointed to the discoordination between the action [vaccination] and information campaigns. He said: “*we first launch an initiative, and the awareness part of it follows later. This awareness becomes a damage control strategy, ie, going for awareness when there is an inertia and stalemate.*”

Another step identified was to uphold the credibility of the vaccination programme. Referring to media reports of expired vials, one educated respondent said, “*I will not expose my children to expired material [vaccines]. It is shameful for our health authorities to allow the use of such things for mass campaigns. I will never feed my children, such drops.*”

A current wave of disinformation on social media by militants is discrediting the Global institutions like the WHO. A propaganda video aired by Tehrek-i-Taliban Pakistan, says that “*In 1972, the World Health Organization published a report requesting that a virus which destroys our immunity T-cells should be incorporated in the vaccines on an experimental basis…Immediately following this experiment, the incidence of AIDS was widely reported in Africa, the Americas, and other countries.*”

Our study sheds light on the community's preference for better service packaging and addressing their overall unmet medical needs. One of our respondents said: “…*why doesn't the government care if a person is dying due to other diseases but concerned about polio?*”

Vaccination does not seem a relevant problem to people when they have unmet basic medical and nutritional needs. A frontline worker said, “*we do not have anything concrete to offer…we do guide them regarding access to primary healthcare or district hospitals. However, when they visit those places and get the inhumane treatment, their gazes for us are furious on our next visit*.”

## DISCUSSION

Our findings suggest that refusals and hesitance are due to contentious issues like sterility, vulgarity, early puberty, the haram composition of vaccines, and using vaccination campaigns as mapping activities for picking drone strikes. These findings support the previous studies, highlighting that vaccination campaigns are believed to be espionage activities [9-13]. As a result of this belief, militant groups carry out active propaganda on social media to discredit vaccination campaigns [14]. Most of our respondents referred to social media propaganda videos as their source of information.

The public health paradigm has shifted from a community perspective to an individual one, and the individual demand needs personalised interaction, ie, targeted information [15,16]. However, our study found that the content of PEI material is silent on many culturally sensitive issues. Due to this lack of targeted awareness, few pockets in Charsadda have developed a strong disliking for vaccines.

There is still an incentive for PEI to create active demand vaccination. Passive acceptance is low yield in the long term [17]. While passive acceptance is higher in populations with low education [18], our findings suggest that highly qualified parents are refusing vaccination now.

Along with refusals, we also interacted with many hesitant parents. These are the parents who have vaccinated their children but still have questions [19]. Evidence suggests that reaching the hesitant parents before they turn to refusals can have promising effects [6,20].

Due to protracted ideological and armed conflict in the region, the Pashtun ethnic group has suffered hugely. This scenario led to an ethnic mistrust and created a public-vs-state confrontation [21]. Given this hostile backdrop, zealous efforts by the state for polio vaccination made the population wary of state's intentions behind it [4]. The negative feelings about vaccination were more severe in the tribal belt and trickled to the rest of Pakistan after the military action ensued against militants [22,23].

Instead of trying to establish a credible image and creating active demand for vaccines, the government has started to arrest parents for refusing vaccination. This step is highly controversial [8]. Our findings suggest that the use of force is counterproductive and further maligns this ethnic group. Some entities have termed these arrests as commendable [24], but our findings suggest otherwise.

The informal Hujra and Jirgah institutions played a vital role in Pashtun society [21]. These institutions dispelled conspiracy theories and inculcated information [25,26]. Our findings suggest that these institutions have collapsed and are being replaced with social media, where malicious messages are spreading at an exponential rate.

The birth of the Digital Hujra (social media) is threatening the lives of vaccinators, the credibility of the Global Polio Eradication Initiative (GPEI) stakeholders and the success of PEI A terrorist organisation (TTP) is effectively using these social media platforms for incitement against polio vaccination. This menace requires immediate redressal. Situations like this can further increase conspiracy and sociological theories [27-29].

With limited government involvement and more foreign influence, the intervention leads to more sociological (conspiracy) theories [30]. Therefore, the institutions and programmes involved with polio eradication need to promote a positive image and strengthen their credibility in the communities.

Our findings also support the fact that there are needs of communities for which they are using polio vaccination as a bargaining chip [31,32]. By fulfilling their basic needs, more active demand for vaccination can be created [17]. Good results are possible with better service packaging and information sharing [22,33]. Through constant, positive engagement, establishing trust and legitimacy, which are the cornerstone for mass-scale interventions, can be achieved [17,34].

### Recommendations

Based on this study, we recommend that the PEI shall engage in open talk to dispel the notions that polio vaccines cause infertility, early puberty, rise in caesarean deliveries, cancer, HIV/AIDS or lead to the birth of more female babies.

The use of force for vaccination should be discouraged. Creation of active-demand through the positive reinforcement of the value of vaccination should be the rule, for which polio vaccination should be presented as part of better service packaging, like the newly launched health insurance programmes.

The PEI and EPI, along with their partners, should fully utilise social media to disseminate accurate information to the public. The propaganda against vaccination on social media must be controlled. The anti-terrorism and cyber-crime laws shall be implemented to take on the militant groups, who use social media to promote refusals, based on false religious notions.

## CONCLUSION

We conclude that vaccine refusals have been persistent over time, but their reasons and manifestations have evolved continuously. The so-called 'religious refusals' are becoming more violent and driven by elements of anti-state sentiments. Refusals rooted in ethnic deprivation are becoming more resistant, invoking administrative actions, further deepening the “us versus them” feeling.

The demise of cultural institutions like Jirga and Hujra is creating a vacuum, which is being filled by social media and its fear mongers. Social media is proving to be the Achilles heel of PEI. Though mainstream awareness and advocacy are needed, engaging social media platforms is vital.

The existing nomenclature for classifying polio refusals is misleading. Active demand through awareness and better packaging of the vaccine within larger benefit schemes like the social health protection (insurance) programme can reduce refusals.
